# Efficient removal of Pb(*II*) and Zn(*II*) ions from aqueous solutions by adsorption onto a native natural bentonite

**DOI:** 10.1016/j.mex.2019.09.005

**Published:** 2019-09-10

**Authors:** Abbas Esmaeili, Hadi Eslami

**Affiliations:** Occupational Environment Research Center, Department of Environmental Health Engineering, School of Health, Rafsanjan University of Medical Sciences, Rafsanjan, Iran

**Keywords:** Application of a native natural bentonite adsorbent for removal of Pb(*II*) and Zn(*II*) ions from aqueous solutions, Adsorption, Natural bentonite, Heavy metals, Lead, Zinc

## Abstract

The new Native Natural Bentonite (NNB) for adsorption of Pb(*II*) and Zn(*II*) ions from aqueous environments was investigated at 27 ± 1^0^C by in batch laboratory experiments. Chemical and mineralogical structure of the NNB adsorbent was characterized by XRF and cation exchange capacity (CEC). The effect of pH, metals concentration, adsorbent dose, and agitation time were also studied. Langmuir and Freundlich isotherm and the Giles classification isotherm were used for describing the equilibrium data. The results show that the NNB contains silica (SiO_2_) and alumina (Al_2_O_3_) as a major chemical compound. The maximum adsorption capacity (mg/g), based on Langmuir isotherm were 8.55 and 7.90 for Pb(*II*) and Zn(*II*), respectively. Pb(*II*) and Zn(*II*) removal efficiency was increasing by increasing the initial pH of solutions, adsorbent dose, and contact time. Therefore, the results of this study show that the equilibrium is reached slowly (180 min), indicating the adsorption sites are not well exposed. By increasing the initial metals ion concentration, the capacity of adsorption decreased and the uptake of Pb(*II*) and Zn(*II*) per unit weight of the adsorbent (mg/g) increased. The adsorption efficiency of Pb(*II*) was higher than Zn(*II*).

## Graphical abstract

**Figure:**
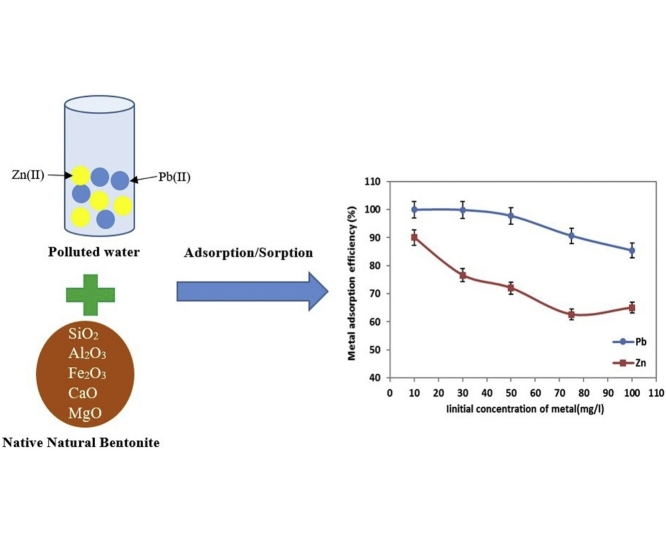

**Specifications Table**

**Table:** 

Subject area:	Environmental Science
More specific subject area:	Adsorption, Heavy metals removal
Protocol name:	Application of a native natural bentonite adsorbent for removal of Pb(*II*) and Zn(*II*) ions from aqueous solutions
Reagents/tools:	A Native Natural Bentonite (NNB) (from Iran) was used as an adsorbent and characterized by XRF. Also, Pb(*II*) and Zn(*II*)concentration were determined by atomic absorption spectroscope (AAS)
Experimental design:	A solution (50 ml) with initial Pb(*II*) and Zn(*II*)concentration (50 mg/L) and pH value (3.0 ± 0.1) was mixed with a definite amount (0.2 to 1.2 g) of NNB. The effect of pH, metals concentration, adsorbent dose, and agitation time were investigated.
Trial registration:	Not applicable
Ethics:	Not applicable

**Value of the Protocol**

**Table:** 

• The results of this study show that the NNB contains silica (SiO_2_) and alumina (Al_2_O_3_) as a major chemical compound and the CEC of NNB was 94.6 meq/100 g. • The maximum adsorption capacity (mg/g), based on Langmuir isotherm were 8.55 and 7.90 for Pb(*II*) and Zn(*II*), respectively and the adsorption efficiency of Pb(*II*) was greater than Zn(*II*). • The data is suitable for removing heavy metals from contaminated water and wastewater.

## Description of protocol

### Preparation of native natural Bentonite

The material used, as adsorbent for this study was a NNB from Sirjan region in Kerman province, located in the south east of Iran. First, the NNB was crushed using jaw crusher (Pulverisette, Fritsch, Germany) then they were sieved. Particles of less than 50 mesh were selected for tests. In addition, the adsorbent was dried for two weeks in the laboratory temperature and air flow [[1], [2], [3]]. Chemical and mineralogical composition of the NNB was identified by X-ray Florescent (XRF) (PHILIPS PW1730, Netherlands). The Cation Exchange Capacity (CEC) of the NNB was measured by Methylene Blue Index, according to the ASTM C 837 -81 [4]. Table 1, Table 2 represent chemical composition, mineralogical structure, and CEC of the NNB adsorbent, respectively. Chemical composition analysis by XRF showed that the NNB contains silica (SiO_2_) and alumina (Al_2_O_3_) as a major compound and other metal oxides, such as Fe_2_O_3_, CaO, MgO, Na_2_O, and K_2_O as minor compound.

**Table 1:** Chemical analysis of dried NNB by XRF analysis.

Composition	Wt%
SiO_2_	64.0
Al_2_O_3_	6.5
Fe_2_O_3_	3.6
CaO	2.2
MgO	3.0
Na_2_O	2.2
K_2_O	0.5
LOI*	10.7

Values were taken from the duplicate samples.

* LOI: Loss of Ignition.

**Table 2:** Mineralogical composition and cation exchange capacity of NNB.

CEC (meq/100 g)	Mineral
94.6	Montmorillonite
	Illite
	Quartz

### Experiment procedure

All chemicals used in this study, are analytical grade reagents which were prepared from Merck Company with ≥99% purity. Adsorption of Pb(*II*) and Zn(*II*) ions from aqueous solutions was studied by the NNB in batch experiments, as a single metal system. All experiments were carried out in 300 ml flat bottom Erlenmeyer flasks with lips at a constant temperature of 27 ± 1 °C using a rotary shaker (150 rpm). A solution (50 ml) with known initial Pb(*II*) or Zn(*II*)concentration (50 mg/L) and pH value (3.0 ± 0.1) was mixed with a definite amount (0.2 to 1.2 g) of NNB and placid in Erlenmeyer flasks. Then the solutions were agitated at lab temperature (27 ± 1 °C) for 300 min with speed of 150 rpm [5,6]. For determining metals concentration in all solutions, atomic absorption spectroscopy (AAS) methods (Varian AA-975 and AA-1275 models, Perkin Elmer) were used. The effect of several parameters, such as initial pH of solutions, concentration of metals, adsorbent dose, and agitation time on sorption of Pb(*II*) and Zn(*II*) ions from aqueous solutions by the NNB was studied. The adsorption capacity of NNB was calculated by applying the following mass balance equation (Eq. (1)):

(1) $$q_{e}=\frac{C_{0}-C_{e}}{m}\times V$$

Where, q_e_ is amount of metal ions absorbed on the NNB (mg/g); C_0_ and C_e_ are the initial and equilibrium concentration (mg/L) of lead and zinc ions, respectively; V is the volume of metal ions solution (L) and m is mass of used adsorbent (g) [7].

### Adsorption isotherms

**Fig. 1:**
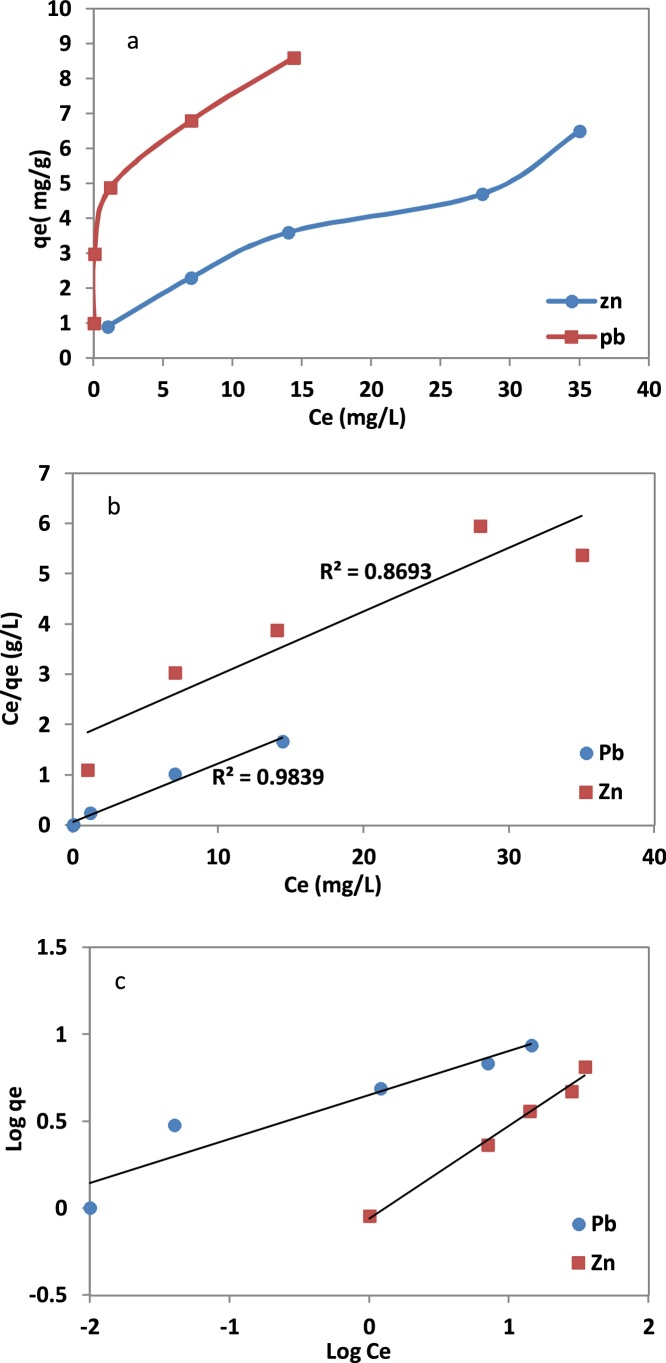
Giles classification (a), Langmuir (b) and Freundlich (c) Isotherms model for adsorption of Pb(II) and Zn(II) ions by NNB.

The adsorption isotherms were studied by mixing 0.5 g of NNB with 50 ml of meal ions of Pb(*II*) and Zn(*II*) solutions at concentrations 10, 30, 50, 75, and 100 mg/L. The pH of solutions was adjusted in 3.0 ± 0.1 with NaOH and HNO_3_ concentrations 1.0 N, then the suspension was placed in rotary shaker and shacked for 300 min with speed of 150 rpm [8]. Langmuir and Freundlich and the Giles classification adsorption isotherms are shown in Fig. 1 and the adsorption parameters are presented in Table 3. Generally, according to the results, the adsorption data of Pb(*II*) and Zn(*II*) on the NNB were fitted well by the Freundlich and Langmuir isotherm equations, respectively. However, satisfactory correlation coefficients were obtained using the later equations for Zn(*II*)(R^2^>0.99) and, Pb(*II*)(R^2^>0.98). In addition, the maximum adsorption capacity of Q_0_ (mg/g), based on Langmuir equation were 8.55 and 7.90 for Pb(*II*) and Zn(*II*), respectively.

**Table 3:** Freundlich and Langmuir model constants for NNB.

Metals	Freundlich adsorption			Langmuir adsorption		
	k_f_	n	R^2^	Q_0_	b	R^2^
Pb(II)	4.11	4.24	0.90	8.55	1.80	0.98
Zn(II)	0.87	1.87	0.99	7.90	0.07	0.87

### The effect of initial concentration of metals

**Fig. 2:**
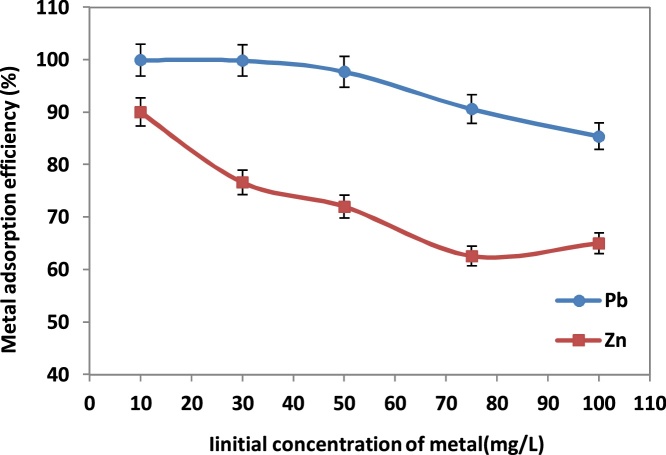
Effect of initial concentration of Pb(II) and Zn(II) ions on the adsorption process.

Fig. 2 shows the effect of Pb(*II*) and Zn(*II*) ions initial concentration on adsorption efficiency of the NNB. The results show when initial metal ions concentration increased, the percentage of adsorption with gentle slopes decreased. Moreover, the results show that about 99% of Pb(*II*) and 90% of Zn(*II*) were removed when initial metal ions concentration was 10 mg/g.

### The effect of adsorbent dosage

**Fig. 3:**
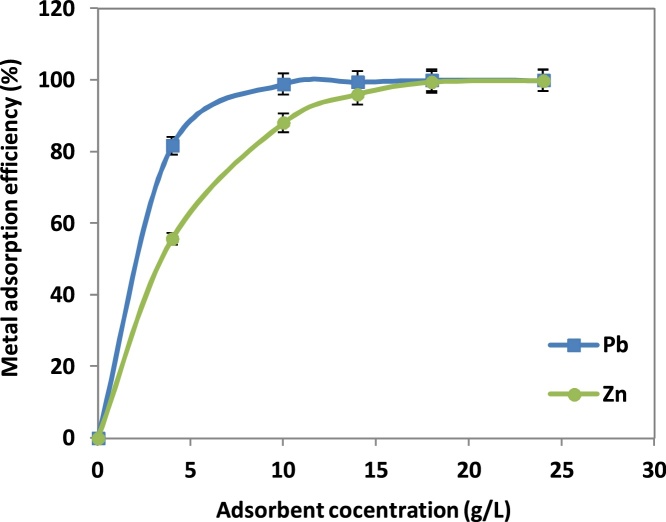
Effect of adsorbent concentration on the adsorption of Pb(II) and Zn(II) ions.

The increasing amount of absorbent increases removal of lead and zinc ions from aqueous solution on the NNB (Fig. 3). This trend was expected; since by increasing sorbent dosage, two phenomena may occur. The first is increasing the adsorption sites; therefore, the adsorption of surface area increased for metal ions [9]. The second is, as the results show in Table 1, the bulk chemical composition of the NNB is alkali and alkaline earth metal oxides.

### The effect of initial pH

**Fig. 4:**
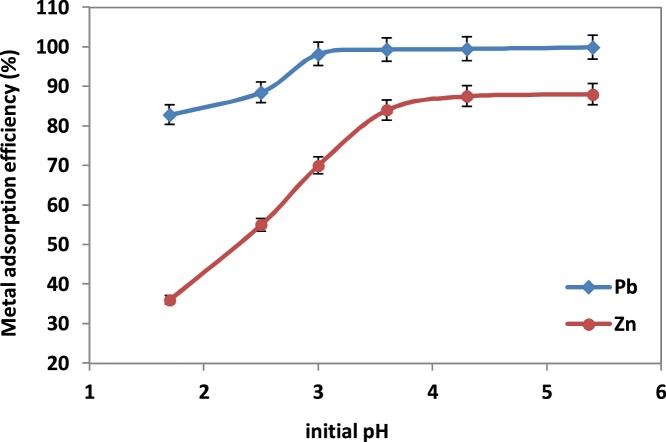
Effect of initial pH on the adsorption process by NNB.

To determine the effect of pH, the tests were carried out using 0.5 g of NNB added to 50 ml Pb(*II*) and Zn(*II*) solutions at concentration 50 mg/L. pH was adjusted in the range of 1.75-5.5. Increasing initial pH of solutions, increase the adsorption of Pb(*II*) and Zn(*II*) (Fig. 4). Based on the results, at low pH the adsorption is negligible, which is typical of metal cations adsorption by natural clay minerals. At low pH, there is a large number of H^+^ ions in the solution which competes with Pb(*II*) and Zn(*II*) ions for active sites on the adsorbent. Consequently, the removal of metals decreases. By increasing pH the number of H^+^ ions reduced and then the competition between H^+^ ions and metal ions for adsorption sites decreased. Therefore, adsorption of lead and zinc ions into NNB increased [2,10,11]. In pH values 2.5 and 3.5 these adsorption percentages increase sharply, reaching almost constant values for higher pH values. The dependence on the sorption effect on pH has almost a S-like course observed by other authors as well in metal cations adsorption with clay minerals [[12], [13], [14]].

### The effect of shaking time

**Fig. 5:**
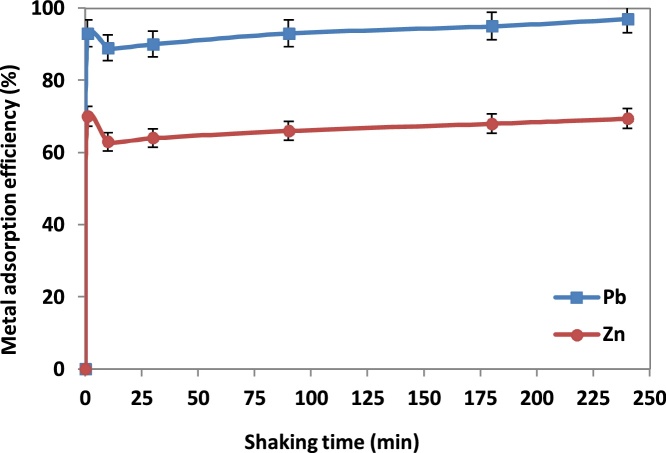
Effect of shaking time on adsorption of Pb(II) and Zn(II) ions by NNB.

The effect of agitation time on the Pb(*II*) and Zn(*II*) ions adsorption by NNB was conducted by varying the agitation time (1–300 min) and using 0.5 g of NNB per 50 ml Pb(*II*) and Zn(*II*) solutions at concentration 50 mg/L. pH was adjusted in 3.0 ± 0.1. Other conditions included particle size of <50 mesh, temperature of 27 ± 1 °C, and shaking rate of 150 rpm. As Fig. 5 reveals the time to reach equilibrium is slowly (180 min), indicating the adsorption sites are not well exposed. Accordingly, there are other mechanisms which likely occurred for the removal of metal ions, such as ion exchange, precipitation, co-precipitation, complex formation, and coagulation- flocculation [12]. This can be considered as one of the advantages of NNB, due to its importance in designing a full scale treatment process.

## Declaration of Competing Interest

There are no conflicts of interests.
